# Influence of tree hollow characteristics and forest structure on saproxylic beetle diversity in tree hollows in managed forests in a regional comparison

**DOI:** 10.1002/ece3.8393

**Published:** 2021-12-08

**Authors:** Benjamin Henneberg, Simon Bauer, Markus Birkenbach, Vanilla Mertl, Manuel J. Steinbauer, Heike Feldhaar, Elisabeth Obermaier

**Affiliations:** ^1^ Department of Animal Ecology I Bayreuth Center of Ecology and Environmental Research (BayCEER) University of Bayreuth Bayreuth Germany; ^2^ Ecological‐Botanical Garden of the University of Bayreuth Bayreuth Germany; ^3^ Department of Sport Ecology Bayreuth Center of Ecology and Environmental Research (BayCEER) University of Bayreuth Bayreuth Germany

**Keywords:** Coleoptera, conservation, dead wood, threatened species, tree hollows, wood mould

## Abstract

Tree hollows are among the rarest habitats in today's Central European managed forests but are considered key structures for high biodiversity in forests. To analyze and compare the effects of tree hollow characteristics and forest structure on diversity of saproxylic beetles in tree hollows in differently structured managed forests, we examined between 41 and 50 tree hollows in beech trees in each of three state forest management districts in Germany. During the two‐year study, we collected 283 saproxylic beetle species (5880 individuals; 22% threatened species), using emergence traps. At small spatial scales, the size of hollow entrance and the number of surrounding microhabitat structures positively influenced beetle diversity, while the stage of wood mould decomposition had a negative influence, across all three forest districts. We utilized forest inventory data to analyze the effects of forest structure in radii of 50–500 m around tree hollows on saproxylic beetle diversity in the hollows. At these larger spatial scales, the three forest management districts differed remarkably regarding the parameters that influenced saproxylic beetle diversity in tree hollows. In Ebrach, characterized by mostly deciduous trees, the amount of dead wood positively influenced beetle diversity. In the mostly coniferous Fichtelberg forest district, with highly isolated tree hollows, in contrast, only the proportion of beech trees around the focal tree hollows showed a positive influence on beetle diversity. In Kelheim, characterized by mixed forest stands, there were no significant relationships between forest structure and beetle diversity in tree hollows. In this study, the same local tree hollow parameters influenced saproxylic beetle diversity in all three study regions, while parameters of forest structure at larger spatial scales differed in their importance, depending on tree‐species composition.

## Graphical abstract

Tree hollows are among the rarest habitats in today's Central European managed forests but are considered key structures for high biodiversity in forests. To analyze and compare the effects of local tree hollow characteristics and forest structure on diversity of saproxylic beetles in tree hollows, we examined between 41 and 50 tree hollows in beech trees in three state forest management districts in Germany. Whereas local tree hollow characteristics seem to similarly influence saproxylic beetle diversity irrespective of region, parameters of forest structure at larger spatial scales differ in their importance, depending on tree‐species composition.

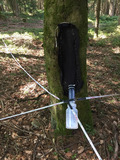

## 1 INTRODUCTION

Recent studies have shown that the abundance and species richness of insects have declined strongly over past decades (Wagner, 2020). This is reported for agricultural landscapes (Wagner, 2020) as well as managed forest ecosystems (Seibold et al., 2019). Intensive forest management has resulted in a massive decline of key elements important for forest biodiversity, such as dead or moribund trees (Vogel et al., 2020). Dead wood is a characteristic and abundant resource in natural forest ecosystems (Seibold & Thorn, 2018) but is often removed in European managed forests (Thorn et al., 2020), resulting in low amounts of dead wood (Gossner, Lachat, et al., 2013; Thorn et al., 2020). Reducing the amount of dead wood in managed forests can help lowering the risk of natural disturbances like wildfires through fuel reduction or pest insect outbreaks (Leverkus et al., 2020). However, this practice also eliminates important habitat features for saproxylic taxa (Thorn et al., 2016). Consequently, many species of saproxylic insects, which directly or indirectly depend on dead wood in at least one stage of their life cycle (Speight, 1989), have become endangered or extinct (Seibold et al., 2015; Thorn et al., 2020). Since approximately 34% of forest‐dwelling species in Europe are regarded as saproxylic (Müller et al., 2008), this also contributes to the decline of forest biodiversity in general. Beetles are especially threatened as >50% of all forest‐dwelling beetle species in Germany are regarded as saproxylic (Köhler, 2000).

Large old trees containing tree hollows are among the rarest structures in European managed forests (Lindenmayer et al., 2012). Tree age and diameter are important factors that facilitate the development of tree hollows (Ranius et al., 2009). In contrast to other dead wood structures like logs, tree hollows provide long‐lasting microhabitats that offer nutritional resources for many species of saproxylic organisms (Siitonen, 2012), and a specific abiotic environment characterized by stable temperature and moisture conditions and increased pH values (Müller et al., 2014). Each tree hollow is unique with regard to the combination of microenvironmental characteristics and the range of microhabitats within (Quinto et al., 2014; Siitonen, 2012). Besides dead wood generalists, tree hollows also provide habitat for many highly specialized saproxylic beetle species (Speight, 1989). These cavity‐dependent tree hollow specialist species complete most of their life cycle in tree hollows and represent an exceptionally threatened group with approximately 75% of them being considered to be threatened or endangered (Schmidl & Büche, 2018).

In past decades European forests were mainly managed for timber production. Since the late 1990s, biodiversity conservation programs with a special focus on dead wood structures have been implemented in an increasing number of European countries, including Finland, Sweden, and Germany (Thorn et al., 2020; Vítková et al., 2018). However, to implement effective conservation measures it is crucial to better understand which forest parameters are the most important to enhance saproxylic beetle diversity. While several studies have analyzed the habitat requirements of saproxylic beetles in general (Müller et al., 2015; Ranius et al., 2015; Seibold et al., 2016), less is known about beetle communities in tree hollows (Micó, 2018; Micó et al., 2015; Quinto et al., 2014; Schauer et al., 2018). Besides limited habitat availability, many saproxylic beetles are assumed to have limited dispersal abilities (Feldhaar & Schauer, 2018). Therefore, it is important to analyze the influence of forest parameters on saproxylic beetle diversity in tree hollows at different spatial scales (Ranius et al., 2015), from characteristics of the tree hollows themselves (Quinto et al., 2014; Schauer et al., 2018), to parameters of forest structure such as distribution of dead wood, age structure, and tree‐species composition (Floren et al., 2014; Gossner et al., 2013; Micó et al., 2013).

The aim of this study was to assess the influence of parameters of tree hollows and the surrounding forest structure on the diversity of saproxylic beetles in tree hollows at different spatial scales. We conducted our study in a regional comparison to achieve generalizable results and to investigate possible regional differences. We selected three state forest regions in Bavaria, Germany, that differed in tree‐species composition but were all representative for Central European managed forests. In addition to local parameters of tree hollows and surrounding forest structure we recorded in the field, we used forest inventory data that are collected systematically for all state forest regions by the Bavarian state forest authority (BaySF) and other German state forest authorities. The use of forest inventory data allowed us to statistically analyze parameters of forest structure at larger spatial scales around the focal tree hollows.

Here we address the following questions: (I) Which parameters of tree hollows and forest structure of managed forests are related to species richness of saproxylic beetles in tree hollows of beech trees at different spatial scales? Based on existing studies we hypothesize that local tree hollow parameters like area of hollow entrance (Quinto et al., 2014) or temperature inside the hollows (Müller et al., 2015; Schauer et al., 2018) as well as early to intermediate stages of wood mould decomposition (Schauer et al., 2018) will positively influence saproxylic beetle species richness, while the amount of dead wood might positively influence beetle species richness at larger spatial scales (Müller et al., 2015). (II) Are there common parameters explaining species richness of hollow‐using beetles across all three forest regions? We hypothesize that local tree hollow parameters might be more influential to saproxylic beetle species richness than parameters of forest structure at larger spatial scales as the quality of tree hollows is crucial for the development of beetle individuals. Tree hollows provide nutritional resources as well as a diversity of microhabitats suitable for the development of saproxylic beetles while parameters of forest structure at larger spatial scales might be more important for accessibility of tree hollows and population sizes of saproxylic beetles.

## 2 MATERIALS AND METHODS

### 2.1 Study sites

The study was conducted in 2018 and 2019 in three Bavarian state forest management districts (Bayerische Staatsforsten, BaySF): Ebrach (N 49°50′, E 10°29′), Fichtelberg (N 49°59′, E 11°50′), and Kelheim (N 48°55′, E 11°52′). These forest management districts were chosen because they represent typical managed forest types in Central Europe as they display the full range of management intensity from strict forest reserves to intensively managed forests (Gossner, Lachat, et al., 2013). The study regions also represent a gradient in tree‐species composition from semi‐natural beech forests (Ebrach) to mixed forests (Kelheim) and forests with a high proportion of planted *Picea abies* trees (Fichtelberg) that is typical for Central European managed forests (Müller et al., 2008).

The forest management district Ebrach in northern Bavaria consists of temperate deciduous forest stands (app. 1000 km^2^, low mountain range, mean annual temperature: 7–8°C, mean annual precipitation: 850 mm [Bässler et al., 2014]). The dominant tree species is beech *Fagus sylvatica* (43% cover), followed by oak (*Quercus robur* and *Quercus petraea*, 20%). Deciduous trees cover more than 70% of the forest area (Müller et al., 2008). The altitude of sample trees ranged from 324 to 482 m a.s.l.

The forest management district Fichtelberg, located in the low mountain range Fichtelgebirge, consists of mainly coniferous forest stands (app. 157 km^2^, mean annual temperature: 5–6°C, mean annual precipitation: 1000–1500 mm [BaySF, 2017]), and is characterized by humid, sub‐alpine climate. The dominant tree species is spruce *P. abies* (80% cover), followed by beech (7%) (BaySF, 2017). The altitude of sample trees ranged from 525 to 873 m a.s.l.

The forest management district Kelheim consists of mixed forest stands (app. 179 km^2^, mean annual temperature: 7–8°C, mean annual precipitation: 650–850 mm [BaySF, 2015]), and is characterized by sub‐oceanic climate. Its forest stands are mixed in tree‐species composition with 56% coniferous and 44% deciduous trees. The dominant tree species is spruce (44% cover), followed by beech (29%) (BaySF, 2015). The altitude of sample trees ranged from 396 to 566 m a.s.l.

**FIGURE A1:**
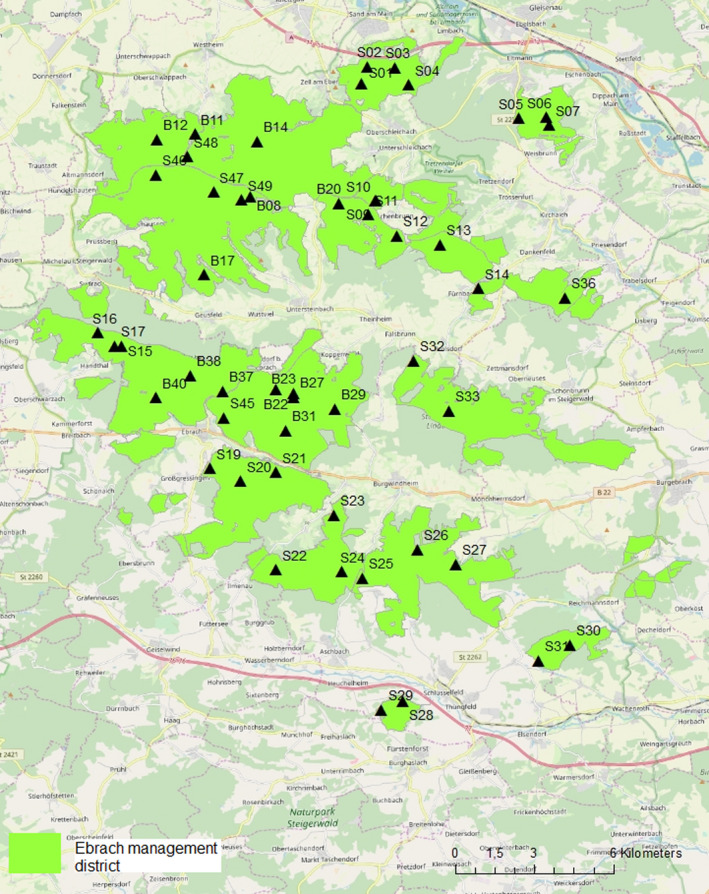
Examined tree hollows in the Ebrach forest management district (*n* = 50)

**FIGURE A2:**
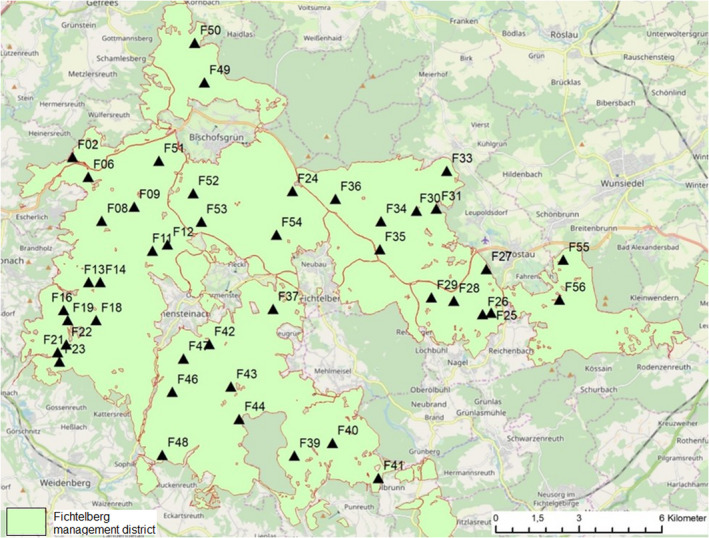
Examined tree hollows in the Fichtelberg forest management district (*n* = 43)

**FIGURE A3:**
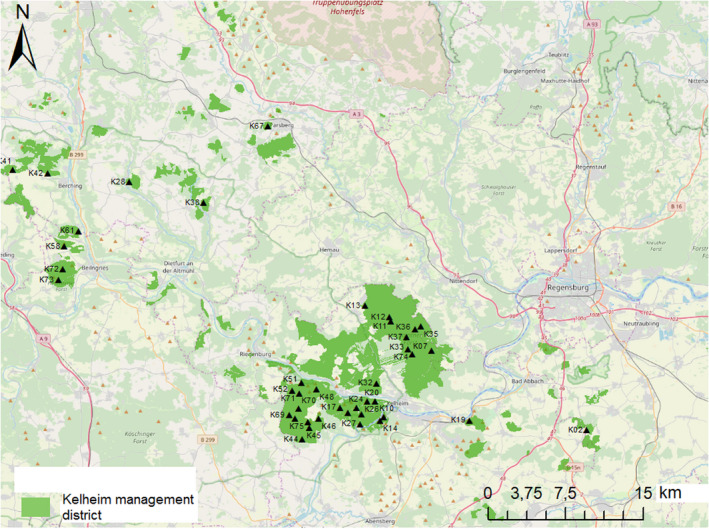
Examined tree hollows in the Kelheim forest management district (*n* = 41)

We selected between 41 and 50 beech trees with tree hollows in each forest management district (Ebrach: 50, Fichtelberg: 43, Kelheim: 41) that were distributed over the whole area of each management district (Figures A1, A2, A3). Tree hollows were selected if they contained at least 2 cm of wood mould at the bottom of the hollow, and the diameter at breast height (DBH) of the host tree was at least 20 cm. Only tree hollows with a maximum height above ground of the lowest point of the hollow entrance of 350 cm were sampled. The minimum distance between two sample trees was 200 m, and the minimum distance to the forest edge was 100 m. We randomly selected tree hollows matching the criteria in each forest stand by assigning each tree hollow in a given stand a number and rolling a dice.

### 2.2 Sampling method and identification of saproxylic beetles

**FIGURE A4:**
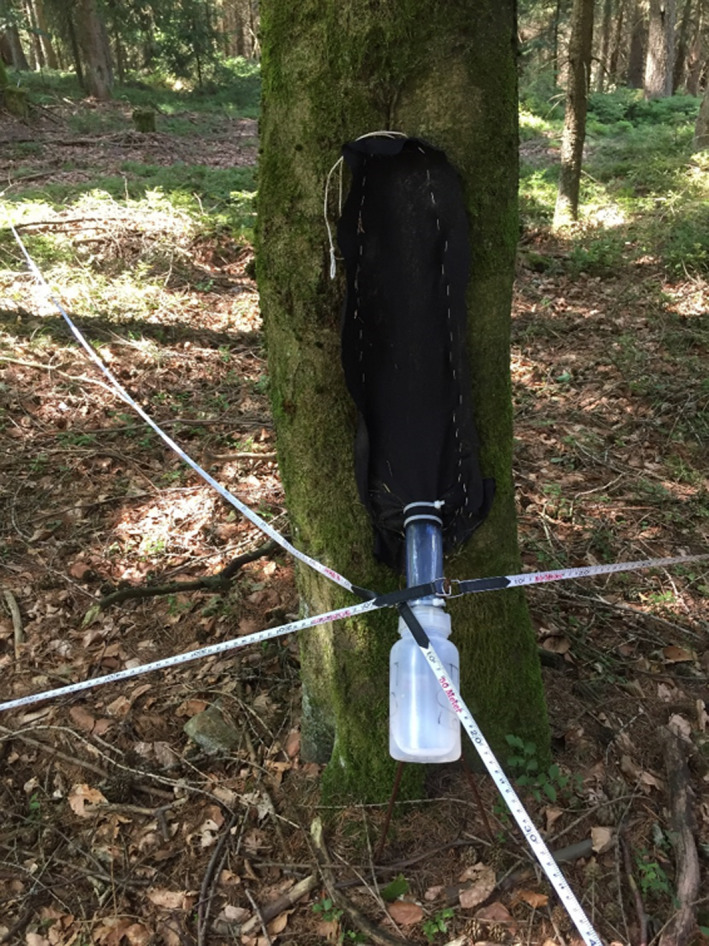
Emergence trap (modified from Gouix & Brustel, 2012)

After selection in February and March (Ebrach/Fichtelberg: 2018, Kelheim: 2019), all tree hollows were closed with black acrylic mesh to prevent vertebrates like birds from using them as nesting place. The black acrylic mesh also did not allow insects to pass. During the sampling period from April to September (18 weeks), all tree hollows were closed with black fabric and emergence traps (modified from Gouix & Brustel, 2012) (Figure A4) that allow efficient sampling of tree hollow arthropod communities as only individuals emerging from the tree hollows will be trapped (Schauer et al., 2018). The collecting bottles contained 99.8% ethanol and were emptied biweekly. A beetle taxonomist (Boris Büche) identified all beetles to species‐level.

### 2.3 Parameters of tree hollows/hollow‐bearing trees recorded in the field

The following parameters of each tree hollow/hollow‐bearing tree were recorded:

- *Area of hollow entrance* calculated as the area of an ellipse with *A* = π*a*b, where *a* is half the height and *b* half the width of the hollow entrance.
- *Hollow volume* calculated as the volume of a cylinder with *V* = π*r ^2^*h, where *r* is the internal radius of the hollow measured at the entrance and *h* the internal height of the hollow measured with a telescopic measuring stick.
- *Height above ground* measured as distance of the lowest point of the hollow entrance to the ground.
- *Diameter at breast height (DBH)* of the hollow tree measured at 130 cm above ground.
- *Height above sea level*: altitude of each tree hollow in m a.s.l.
- *Stage of decomposition*: the stage of decomposition of wood mould sampled from the base of each tree hollow at a depth of 2–5 cm, using a spoon that was attached to a stick, was determined using three parameters, color, texture, and visible woody parts, and was classified in four ascending categories according to Jarzabek (2005):
*Stage 1/low decay:* yellow to light brown in color, visible woody parts of bigger size.
*Stage 2/medium decay:* light brown to brown in color, visible woody parts of smaller size.
*Stage 3/medium to high decay:* brown to dark brown in color, almost no visible woody parts.
*Stage 4/high decay:* dark brown to black in color, no visible woody parts.
- *Temperature inside the hollow* measured with temperature loggers inside each tree hollow every 60 min over the whole sampling period.

### 2.4 Parameters of forest structure recorded in the field

Two parameters of forest structure were recorded around each tree hollow:

- *Surrounding microhabitat structures*: the number of microhabitat structures in trees according to Kraus et al. (2016) in a 30 m radius around each tree hollow: woodpecker holes, visible tree fungi, broken branches with a minimum diameter of 12 cm, and injuries to the bark with a minimum area of 250 cm^2^. These tree‐related microhabitat structures have been widely recognized as important substrates and structures for saproxylic biodiversity in forests and are receiving increasing attention in management, conservation and research (Larrieu et al., 2018).
- *Surrounding tree hollows*: the number of visible tree hollows in a 30 m radius around each tree hollow.

### 2.5 Parameters of forest structure assessed via forest inventory data

We used forest inventory data collected by the Bavarian state forest authorities (BaySF) in 2010–2012. Sampling of forest inventory data is conducted every 10 years by the BaySF on a 200 × 200 m grid over the whole area of the forest management district, with a sampling point at each nodal point. More than 100 parameters are recorded within a parameter‐dependent radius around each sampling point, out of which we chose those that we expected to influence saproxylic beetle diversity: *amount of dead wood* (volume of all dead woody parts, standing or downed, with a minimum diameter of 20 cm), sampled within a radius of 12.62 m (500 m^2^) around each sampling point, and *DBH of deciduous trees* (the mean diameter at breast height in cm) as a proxy for age structure of deciduous trees, also sampled within a radius of 12.62 m (500 m^2^) around each sampling point. Additionally, in the forest management district Fichtelberg that consisted mainly of coniferous trees, we examined the *proportion of beech trees*. For parameters *DBH of deciduous trees* and *proportion of beech trees*, only trees assigned to the upper forest layer were included in the analysis to avoid overrepresentation of young trees that lack suitable microhabitats for saproxylic beetles.

### 2.6 Interpolation of forest inventory data

**FIGURE A5:**
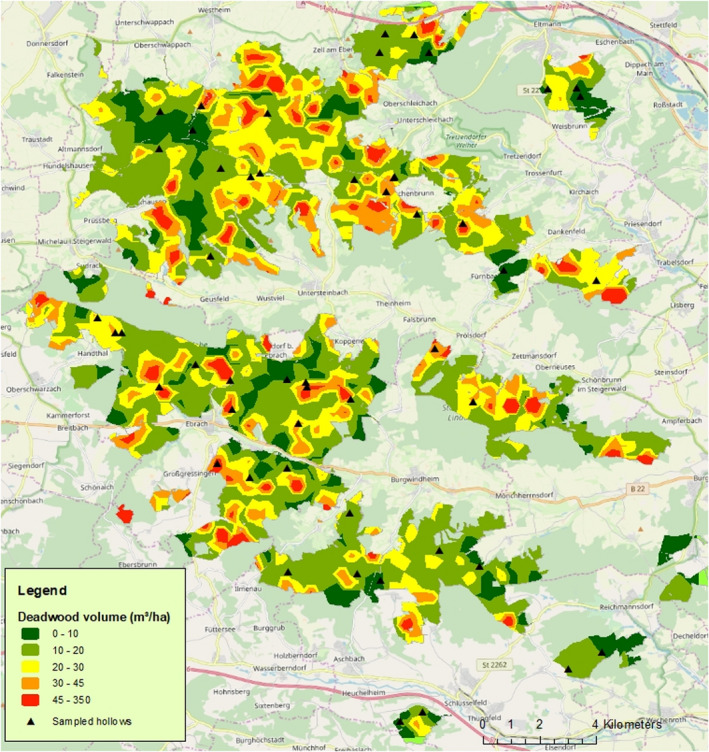
Distribution of dead wood in the Ebrach forest management district; map generated from forest inventory data using the deterministic inverse distance weighting (IDW) method

**FIGURE A6:**
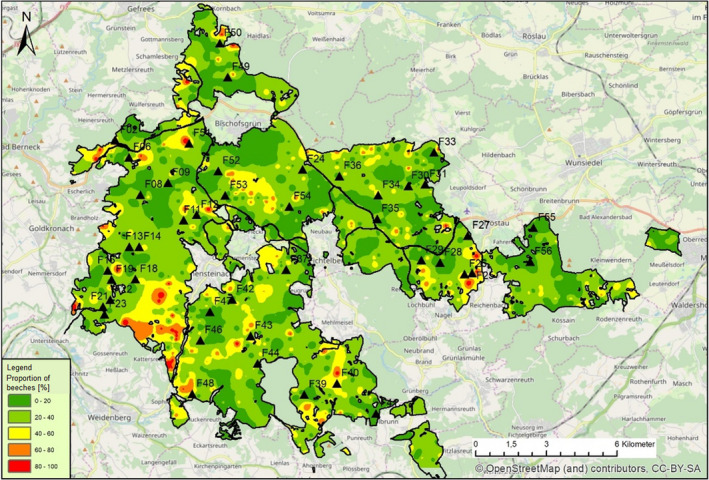
Proportion of beech trees in the Fichtelberg forest management district; map generated from forest inventory data using the deterministic inverse distance weighting (IDW) method

Because forest inventory data is recorded as point data, it must be interpolated to maps with a continuous spatial distribution prior to regression analysis with species richness of saproxylic beetles. We tested the forest inventory parameters for spatial autocorrelation by visually inspecting semivariograms in the software ArcGIS (ESRI, 2018). The only parameter of forest inventory data where we detected spatial autocorrelation and that also had a normal distribution, and we therefore were able to apply the geostatistical Kriging approach was *DBH of deciduous trees* in the forest management district Ebrach. For all other forest inventory parameters and forest management districts we applied the deterministic inverse distance weighting (IDW) method to spatially interpolate forest inventory data and create interpolation maps (Figures A5, A6).

### 2.7 Statistical analysis of forest inventory data

Interpolation maps displaying forest inventory parameters cannot be directly used for statistical analysis as they display a spatially continuous data distribution. To obtain values for each sampled tree hollow we used the software ArcGIS (ESRI, 2018) to transform the interpolated data to a point grid of 10 × 10 m. We drew circular buffers around each tree hollow with radii ranging from 50 to 500 m and calculated the average value for each forest inventory parameter within each buffer. We used these values for the statistical analysis to examine the forest inventory parameters at different spatial scales around each tree hollow. We randomly excluded buffer sizes or single tree hollows from the analysis when there was more than 10% overlap of the buffer areas of neighboring tree hollows or with the border of the forest management district.

### 2.8 Statistical analysis

To visualize similarities in species composition between the three study sites, detrended correspondence analysis (DCA) was performed using the R function *decorana* in the *vegan* package (Oksanen et al., 2013). To analyze the influence of tree hollow parameters and parameters of forest structure on species richness of hollow‐using beetles, generalized linear models (GLMs) with Poisson error distribution were implemented. Collinearity among explanatory variables was tested using Pearson's linear correlation (exclusion criterion for two variables in the same model |*r*| > .7). Univariate GLMs were used for preselection of explanatory variables for multivariate GLMs. All explanatory variables with significant influence on species richness in univariate GLMs were used in combination with forest inventory data in multivariate GLMs. If two variables showed collinearity they were used in separate models. If there were no local tree hollow parameters with significant influence on species richness in univariate GLMs, the four parameters with the lowest *p*‐values were selected for multivariate GLMs. To improve model fitting and the distribution of residuals all explanatory variables were transformed. Depending on the data distribution, either a log‐ or sqrt‐transformation was chosen. By visually inspecting the distribution of residuals in univariate GLMs the model fit of transformed variables was compared to the untransformed variables, and the version that provided the best distribution of residuals was selected for multivariate GLMs. Stepwise model selection based on AIC was used to remove non‐relevant variables from the models (R function *step* in the *stats* package). All multivariate models were tested for overdispersion using the R function *dispersion*.*test* in the *AER* package (Kleiber & Zeileis, 2008). Each explanatory variable that showed significant influence on species richness in multivariate GLMs was visualized using the R package *visreg* (Breheny & Burchett, 2017). Analyses were performed for total species richness of saproxylic beetles as well as for the subset of threatened species (i.e. species listed with a status of 0–3 on the Red List of Germany (Schmidl & Büche, 2018)).

To combine all three forest management districts in a single model we created generalized linear mixed‐effects models (GLME) using the R function *glmer* in the *lme4* package (Bates et al., 2015), with parameters of tree hollows and forest structure as fixed factors and forest management district as the random factor. Residuals of random factors were examined visually by inspecting the fitted vs. residuals plot (R function *plot* in the *lme4* package [Bates et al., 2015]) and the residuals vs. predicted plot (R function *simulateResiduals* in the *DHARMa* package [Hartig, 2020]). All analyses were performed with the software R, version 3.6.3 (R Core Team, 2020).

## 3 RESULTS

### 3.1 Saproxylic beetles

In the three forest management districts (134 tree hollows), we collected a total of 5880 saproxylic beetle individuals, belonging to 283 species and 48 families. Sixty‐two species (21.9%) are regarded as threatened (Schmidl & Büche, 2018). Four species of the collected beetle species are critically endangered (Red List of Germany status 1 [Schmidl & Büche, 2018]): *Ampedus brunnicornis*, *Crepidophorus mutilatus*, *Cryptophagus deubeli*, *Prionychus melanarius*. The sampled species are very diverse regarding body size (ranging from 1–2 mm to approximately 5 cm) and ecological guild (Schmidl & Bußler, 2004). Only few species are flightless; the majority is mobile. However, for most species it is not known if they are highly mobile or more or less sessile, and how far they disperse within a forest or even between forest regions (Feldhaar & Schauer, 2018).

**FIGURE 1:**
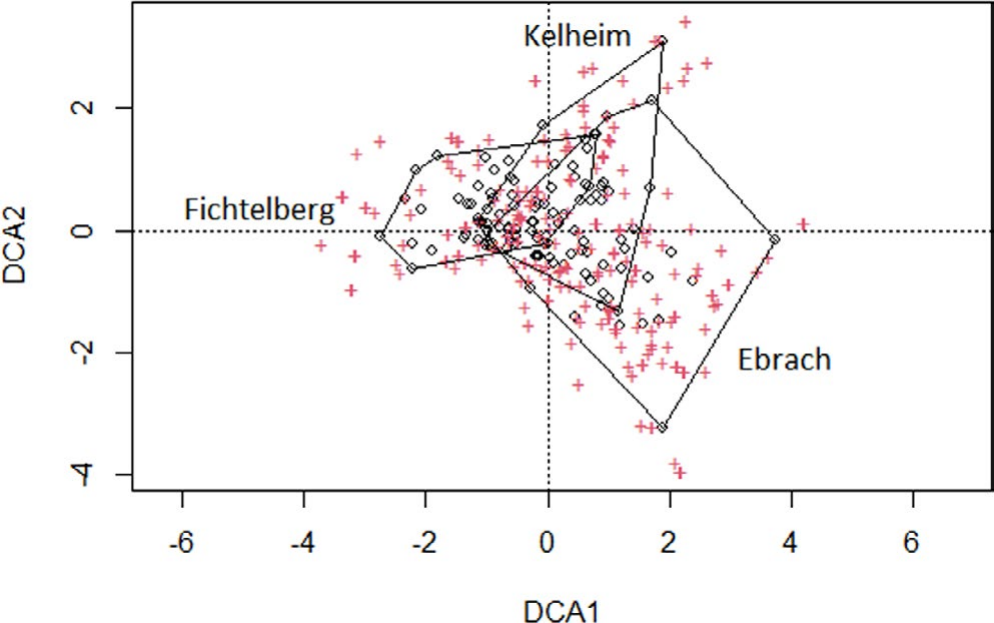
Detrended correspondence analysis (DCA) that visualizes similarities in species composition between the three study sites; circles = tree hollows, crosses = saproxylic beetle species

In 2018 we collected 4151 individuals in Ebrach (50 tree hollows), belonging to 196 species from 43 families with 41 species (20.9%) being regarded as threatened. The same year we collected 441 individuals in Fichtelberg (43 tree hollows), belonging to 74 species from 24 families with nine species (12.2%) being regarded as threatened. In 2019 we collected 1288 individuals in Kelheim (41 tree hollows), belonging to 107 species from 32 families with 28 species (26.2%) being regarded as threatened. The average species richness of saproxylic beetle species per tree hollow was 13.2 ± 6.0 (mean ± SD) in Ebrach, 4.6 ± 2.7 in Fichtelberg, and 7.9 ± 3.7 in Kelheim. Similarities in species composition between the three study sites were visualized using detrended correspondence analysis (DCA) (Figure 1).

### 3.2 Effects of tree hollow characteristics and forest structure on saproxylic beetle diversity

#### 3.2.1 Forest management district Ebrach

**FIGURE 2:**
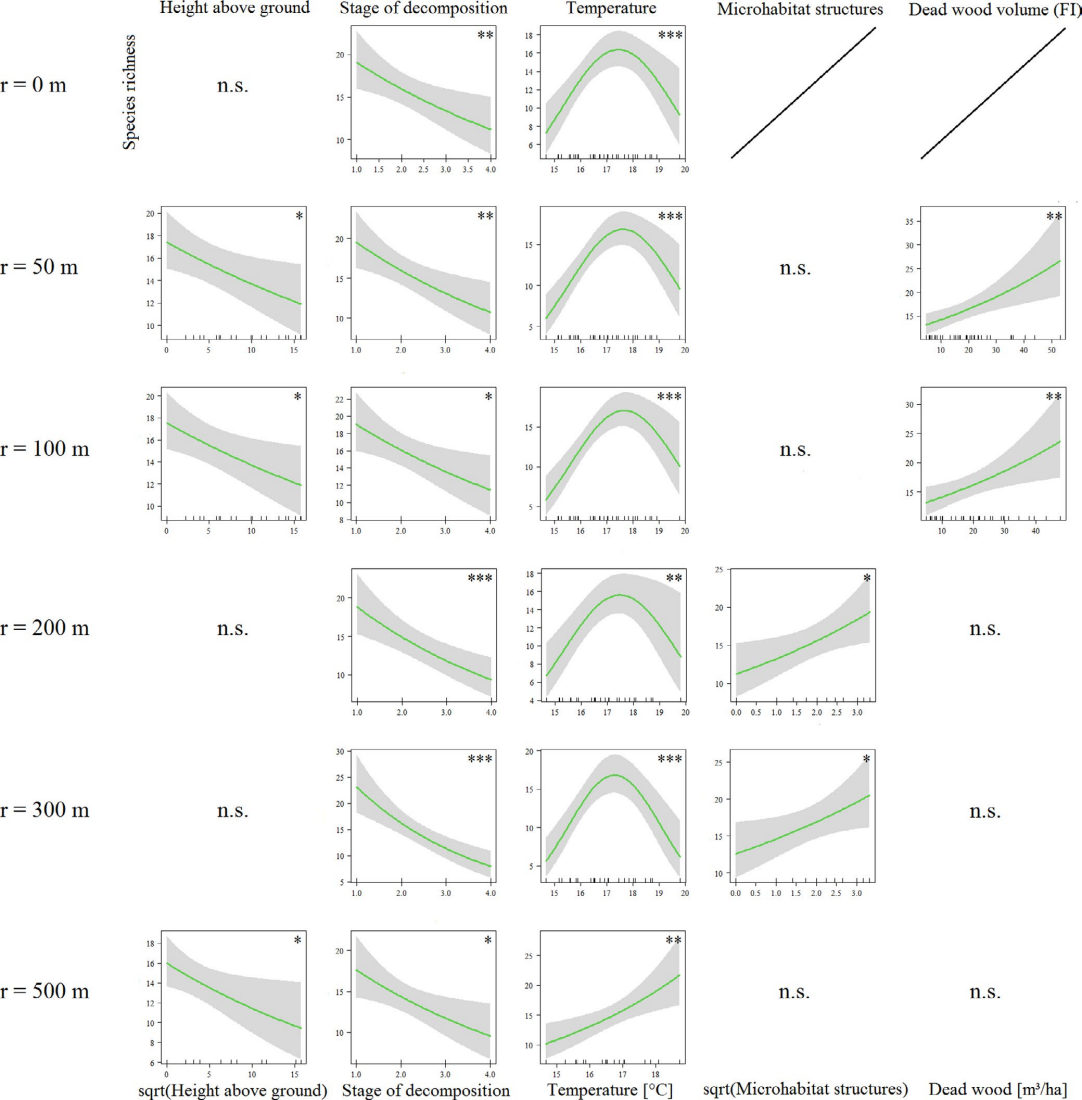
Multivariate GLMs of the Ebrach management district (2018) of different radii around the focal tree hollows (*r* ≤ 100 m: *n* = 50, *r* = 200 m: *n* = 37, *r* = 300 m: *n* = 32, *r* = 500 m: *n* = 24). Total species richness of saproxylic beetles as dependent variable; *p* < .05 (*), *p* < .01 (**), *p* < .001 (***)

In Ebrach species richness of saproxylic beetles was negatively related to the height of the tree hollows above ground (*z* = −2.31, *p* < .05), and to the *stage of decomposition* of the wood mould inside the hollows (*z* = −2.82, *p* < .001). There was a hump‐shaped relationship to *temperature* inside the hollows (*z* = 3.82, *p* < .001). This relationship turned positive in the *r* = 500 m model. Furthermore, representing forest structure at larger spatial scales, the number of microhabitat structures in a 30 m radius around the tree hollows (*surrounding microhabitat structures*, *z* = 2.32, *p* < .05), and the amounts of dead wood up to a radius of 100 m around the tree hollows (*dead wood volume*, *r* = 50 m, *z* = 3.24, *p* < .01; *r* = 100 m, *z* = 2.72, *p* < .01) were positively correlated with the total number of saproxylic beetle species in the tree hollows (Figure 2, Table A1). Pseudo‐*r* ^2^ values that show the explanatory power of the models range from 0.378 to 0.740 depending on radius (Table A1).

**FIGURE A7:**
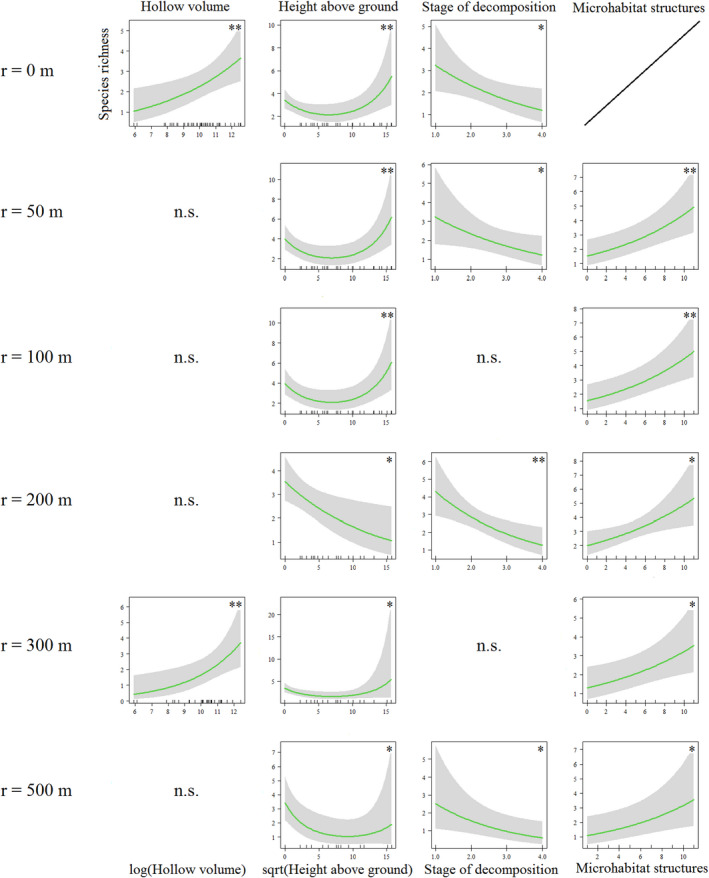
Multivariate GLMs of the Ebrach management district (2018) of different radii around the focal tree hollows (*r* ≤ 100 m: *n* = 50, *r* = 200 m: *n* = 37, *r* = 300 m: *n* = 32, *r* = 500 m: *n* = 24). Species richness of threatened saproxylic beetles as the dependent variable; *p* < .05 (*), *p* < .01 (**), *p* < .001 (***)

When only the 41 threatened saproxylic beetle species were included in the analysis, the volume of the tree hollows (*z* = 3.21, *p* < .01) was the only local tree hollow parameter that was positively correlated with the number of beetle species. The height of the hollow entrance above ground (*z* = −2.60, *p* < .01) and the *stage of decomposition* of the wood mould (*z* = −2.70, *p* < .01) were negatively correlated with the number of species. The number of microhabitat structures in a 30 m radius around the tree hollows (*surrounding microhabitat structures*, *r* = 30 m, *z* = 3.24, *p* < .01) was the only parameter of forest structure that was positively correlated with the number of threatened saproxylic beetle species (Figure A7, Table A2). Pseudo‐*r* ^2^ values that show the explanatory power of the models range from 0.288 to 0.691 depending on the radius (Table A2).

#### 3.2.2 Forest management district Fichtelberg

**FIGURE 3:**
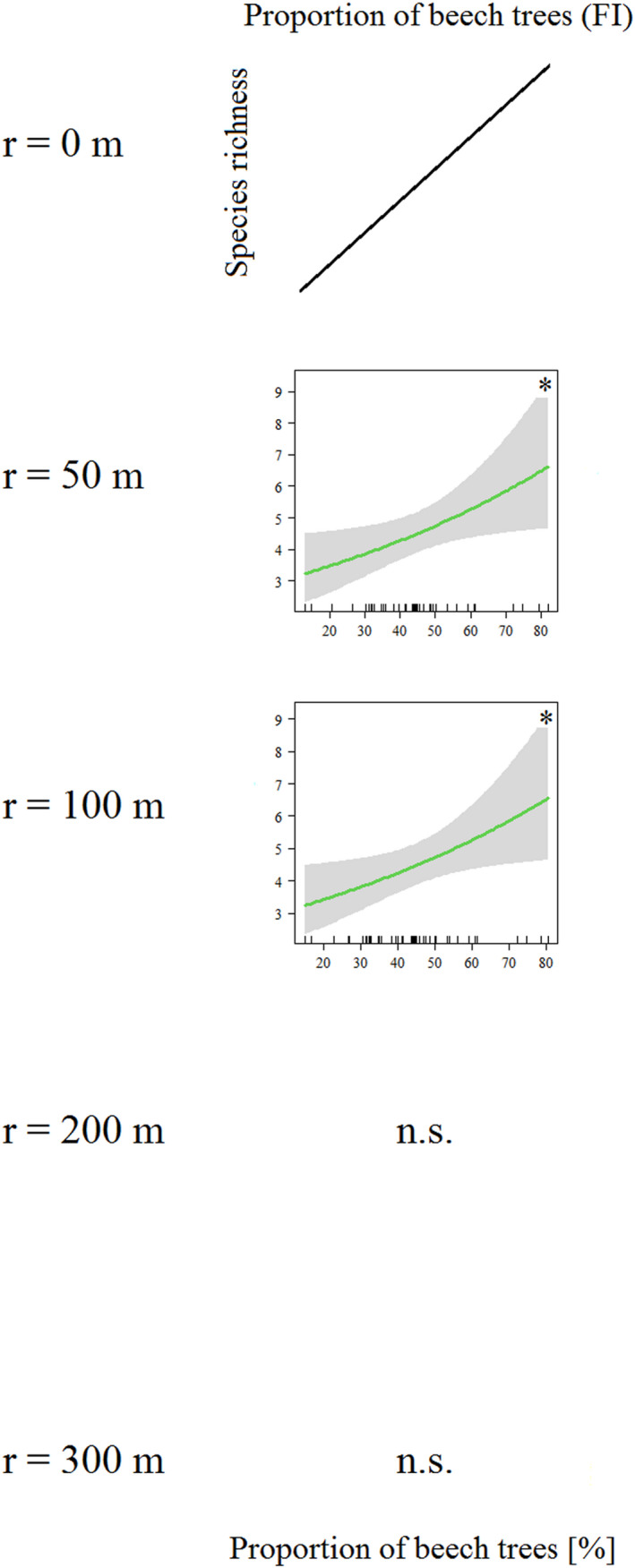
Multivariate GLMs of the Fichtelberg management district (2018) of different radii around the focal tree hollows (*r* ≤ 100 m: *n* = 43, *r* = 200 m: *n* = 36, *r* = 300 m: *n* = 33, *r* = 500 m: *n* = 28). Total species richness of saproxylic beetles as the dependent variable; *p* < .05 (*), *p* < .01 (**), *p* < .001 (***)

In Fichtelberg species richness of saproxylic beetles was positively related only to the proportion of beech trees in the tree‐species composition at different spatial scales around the tree hollows (*proportion of beech trees*, *r* = 50 m, *z* = 2.27, *p* < .05; *r* = 100 m, *z* = 2.28, *p* < .05) (Figure 3, Table A3). Thus, with increasing proportion of beech trees in the surrounding of tree hollows, the total number of hollow‐using beetle species increased in this conifer dominated forest area. Pseudo‐*r* ^2^ values that show the explanatory power of the models are 0.113 (*r* = 50 m) and 0.116 (*r* = 100 m) (Table A3).

**FIGURE A8:**
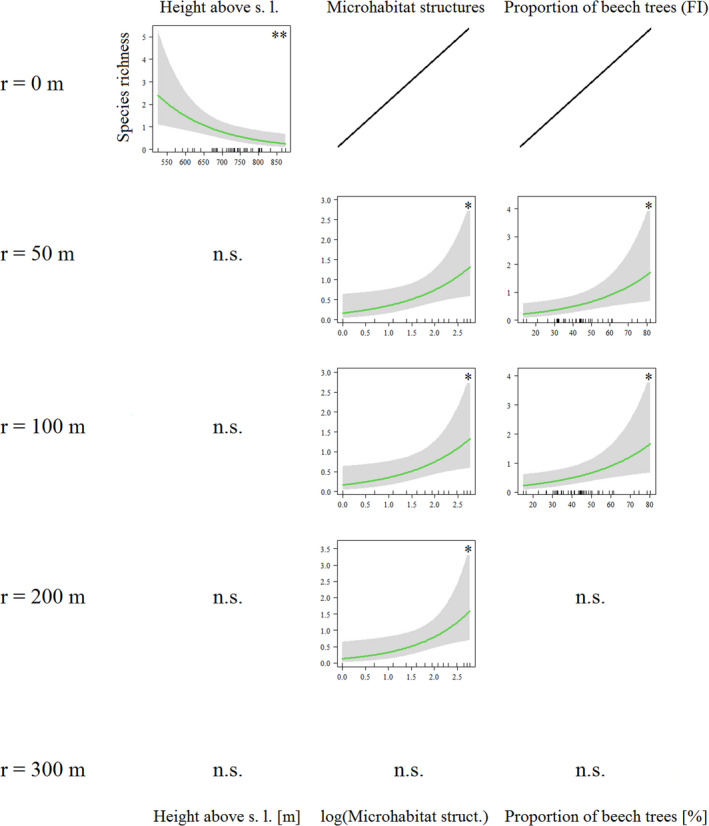
Multivariate GLMs of the Fichtelberg management district (2018) of different radii around the focal tree hollows (*r* ≤ 100 m: *n* = 43, *r* = 200 m: *n* = 36, *r* = 300 m: *n* = 33, *r* = 500 m: *n* = 28). Species richness of threatened saproxylic beetles as the dependent variable; *p* < .05 (*), *p* < .01 (**), *p* < .001 (***)

When only the nine threatened saproxylic beetle species were included in the analysis, species richness was positively related to the number of microhabitat structures in a 30 m radius around the tree hollows (*surrounding microhabitat structures*, *r* = 30 m, *z* = 2.19, *p* < .05) and again the *proportion of beech trees* up to a radius of 100 m around the focal tree hollows (*r* = 50 m, *z* = 2.54, *p* < .05; *r* = 100 m, *z* = 2.50, *p* < .05) (Figure A8, Table A4). Pseudo‐*r* ^2^ values that show the explanatory power of the models range from 0.203 to 0.487 depending on radius (Table A4).

#### 3.2.3 Forest management district Kelheim

**FIGURE 4:**
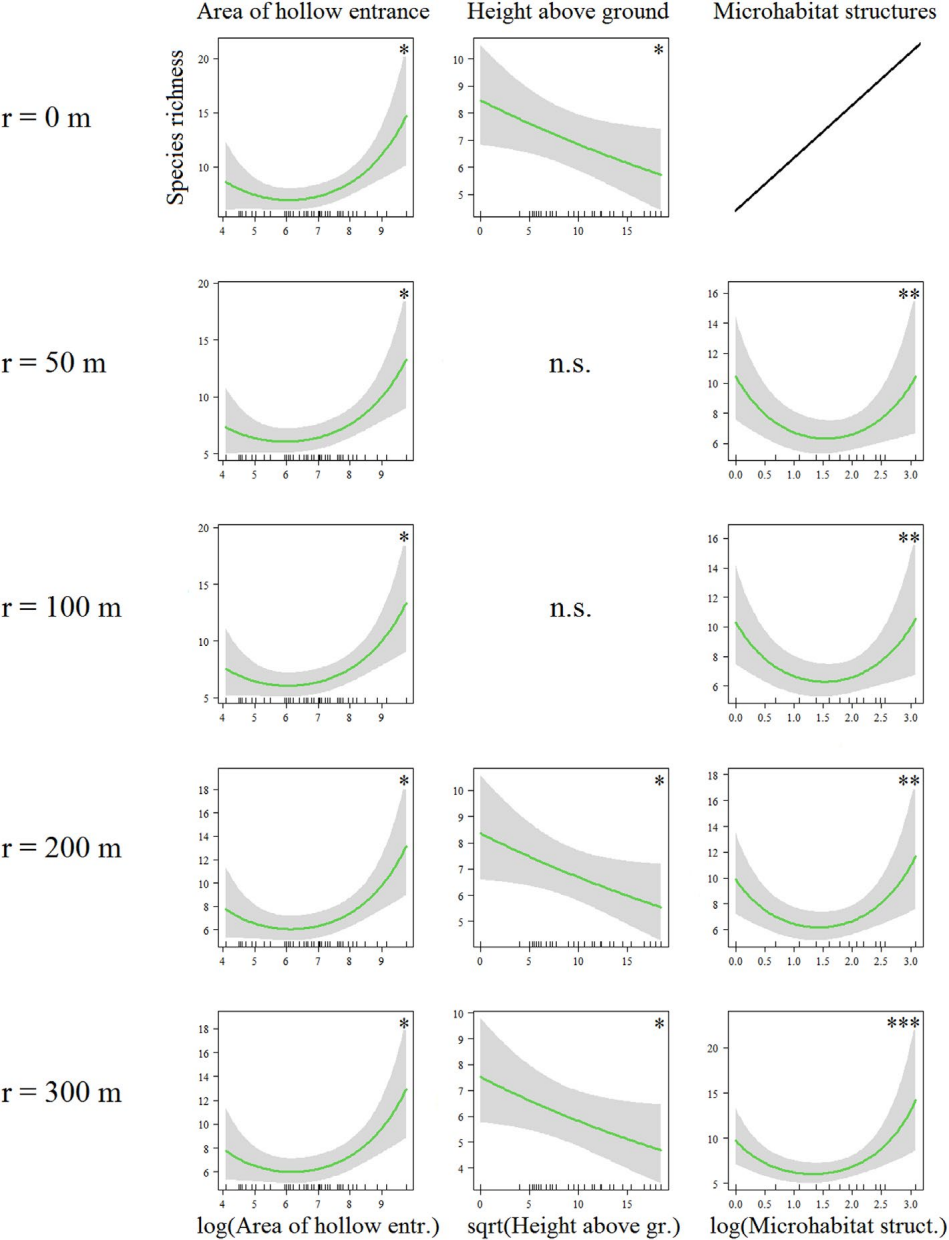
Multivariate GLMs of the Kelheim management district (2019) of different radii around the focal tree hollows (*r* ≤ 100 m: *n* = 41, *r* = 200 m: *n* = 35, *r* = 300 m: *n* = 32). Total species richness of saproxylic beetles as the dependent variable; *p* < .05 (*), *p* < .01 (**), *p* < .001 (***)

In Kelheim species richness of saproxylic beetles showed a U‐shaped relationship to the size of the *area of hollow entrance* (*z* = −2.23, *p* < .05). This can be explained by a single tree hollow with a small entrance area. When that hollow was removed from the analyses, the relationship became positive. There was a negative relationship between species richness and height of the hollow entrance above ground (*z* = −1.96, *p* < .05). The number of microhabitat structures in a 30 m radius around the tree hollows (*surrounding microhabitat structures*, *r* = 30 m, *z* = −3.16, *p* < .01) also showed a U‐shaped relationship to total species richness (Figure 4, Table A5). Pseudo‐*r* ^2^ values that show the explanatory power of the models range from 0.240 to 0.434 depending on the radius (Table A5).

**FIGURE A9:**
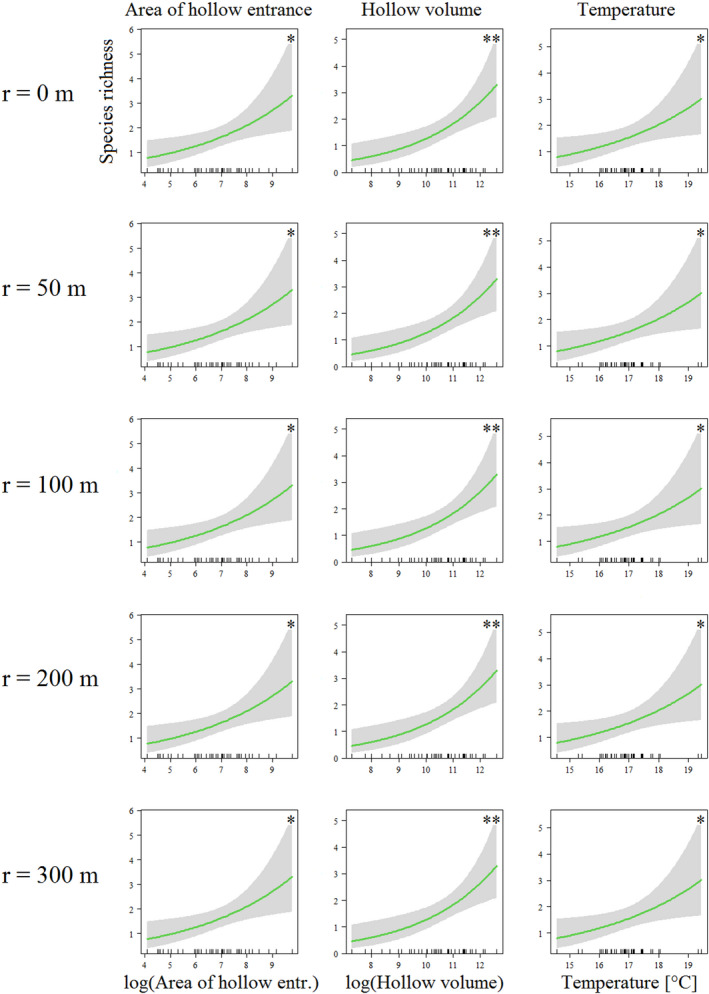
Multivariate GLMs of the Kelheim management district (2019) of different radii around the focal tree hollows (*r* ≤ 100 m: *n* = 41, *r* = 200 m: *n* = 35, *r* = 300 m: *n* = 32). Species richness of threatened saproxylic beetles as the dependent variable; *p* < .05 (*), *p* < .01 (**), *p* < .001 (***)

Species richness of 28 threatened saproxylic beetle species was positively related to the volume of the tree hollows (*z* = 3.21, *p* < .01), the *area of hollow entrance* (*z* = 2.54, *p* < .05) and the *temperature* inside the hollows (*z* = 2.28, *p* < .05) (Figure A9, Table A6). As there were no statistically significant parameters of forest structure, Pseudo‐*r* ^2^ that shows the explanatory power of the models is 0.293 for all radii (Table A6).

#### 3.2.4 Combined analysis of the three forest management districts

**FIGURE 5:**
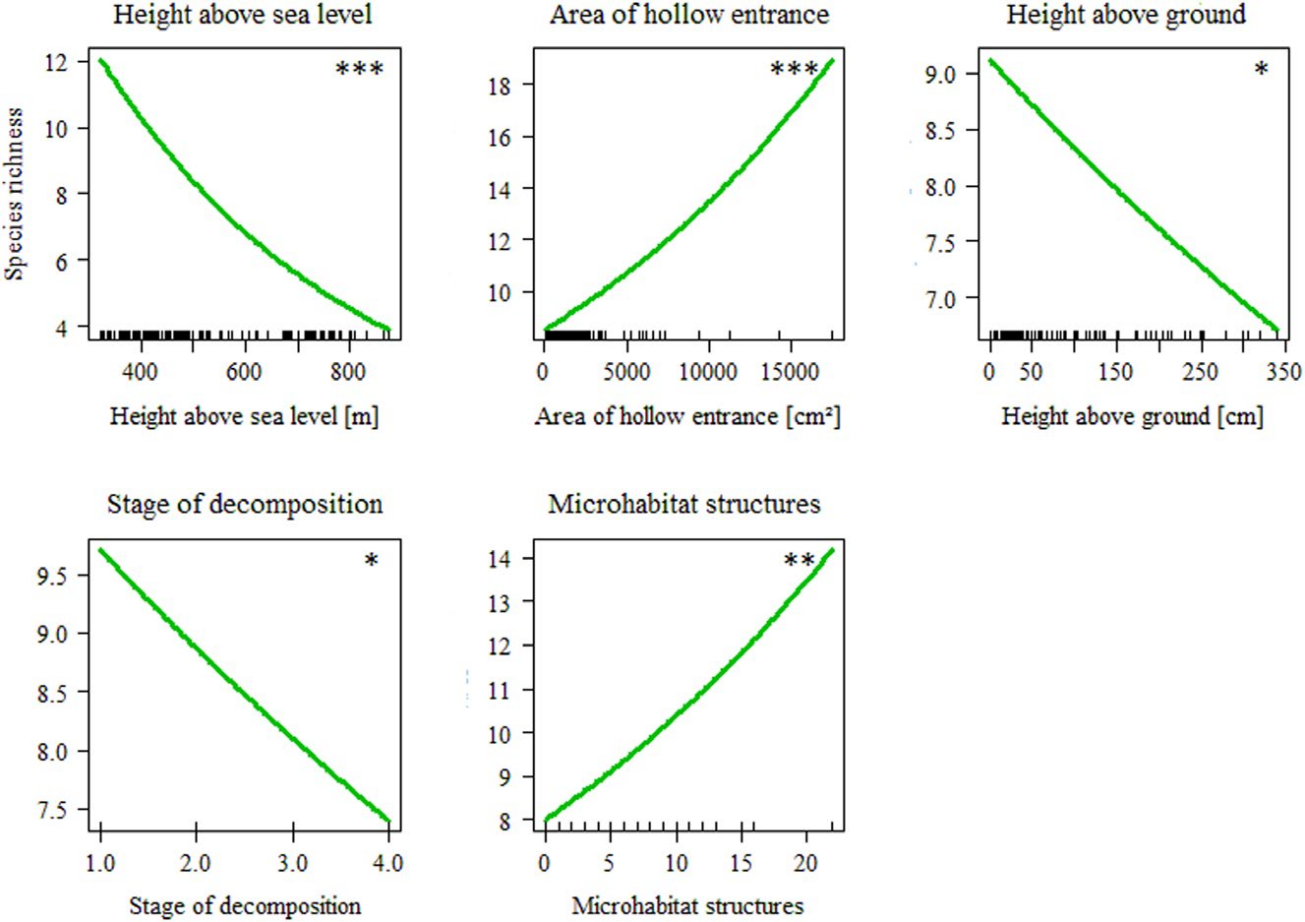
Generalized linear mixed effects models (GLMEs) of the three forest management districts combined (*n* = 134 tree hollows). Total species richness of saproxylic beetles as the dependent variable; *p* < .05 (*), *p* < .01 (**), *p* < .001 (***)

Species richness of saproxylic beetles over all three management districts combined was negatively related to local tree hollow parameters *height above sea level* (*z* = −4.45, *p* < .001), height of the hollow entrance above ground (*z* = −2.15, *p* < .05), and the *stage of decomposition* of the wood mould (*z* = −2.19, *p* < .05), and positively related to the *area of hollow entrance* (*z* = 4.17, *p* < .001). The only parameter of forest structure that explained total species richness across all three management districts was the number of microhabitat structures in a 30 m radius around the tree hollows (*surrounding microhabitat structures*, *z* = 2.70, *p* < .01) (Figure 5, Table A7).

**FIGURE A10:**
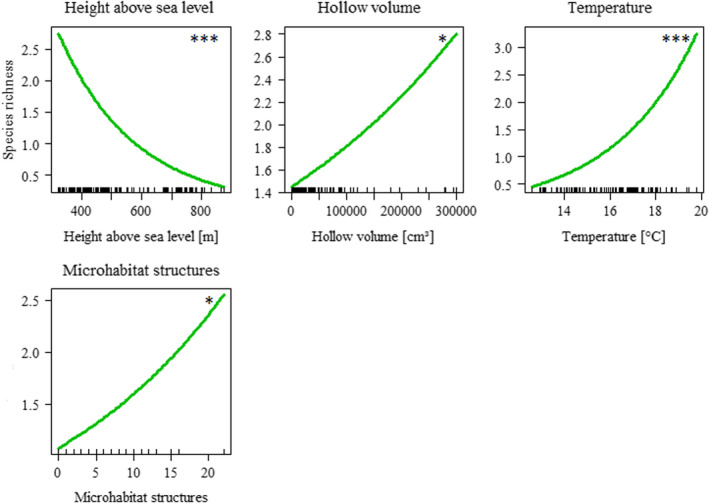
Generalized linear mixed effects models (GLMEs) of the three forest management districts combined (*n* = 134 tree hollows). Species richness of threatened saproxylic beetles as the dependent variable; *p* < .05 (*), *p* < .01 (**), *p* < .001 (***)

When only the 61 threatened saproxylic beetle species were included in the analysis, species richness was positively related to the volume of the tree hollows (*z* = 2.18, *p* < .05), and the *temperature* inside the hollows (*z* = 5.37, *p* < .001), and negatively related to *height above sea level* (*z* = −5.47, *p* < .001). The only parameter describing forest structure that explained species richness of threatened saproxylic beetles across all three management districts was again the number of microhabitat structures in a 30 m radius around the tree hollows (*surrounding microhabitat structures*, *z* = 2.01, *p* < .05) (Figure A10, Table A8).

## 4 DISCUSSION

In this study, the influence of local tree hollow parameters as well as the surrounding forest structure within three managed forests on species richness of saproxylic beetles in tree hollows was investigated. Our results confirm the findings that species richness of hollow‐using saproxylic beetles is related on the one hand to different properties of the tree hollows themselves (Micó et al., 2015; Quinto et al., 2014; Schauer et al., 2018): when all three forest regions were combined the area of hollow entrance positively influenced total species richness while height above ground and stage of decomposition negatively influenced total species richness of saproxylic beetles; on the other hand, the influence of these local properties of tree hollows on species richness of saproxylic beetles also depends on surrounding forest structure. In forest regions with a higher proportion of deciduous trees (Ebrach, Kelheim) the influence of local tree hollow parameters was more pronounced than in the mostly coniferous Fichtelberg forest region. When regions were analyzed separately, the only parameter (of those investigated) that influenced total species richness of saproxylic beetles in the conifer‐dominated Fichtelberg region was the proportion of beech trees in the surrounding of focal tree hollows. Beech trees in this forest region can be described as isolated within a matrix of coniferous trees which made their presence the most important resource for saproxylic beetles under these circumstances. Therefore, the influence of local tree hollow characteristics on diversity of saproxylic beetles must be seen in relation to surrounding forest structure. However, the sample size is potentially limited for the analysis of each region separately, as there were only 41–50 tree hollows examined in each forest region.

### 4.1 Influence of local tree hollow parameters

One local tree hollow parameter that showed a positive relationship to species richness when all three forest regions were combined was area of hollow entrance. A positive relationship with area of hollow entrance was also observed for threatened species in Kelheim. This relationship has also been observed in previous studies (Quinto et al., 2014; Ranius, 2002; Schauer et al., 2018). It has been proposed that many saproxylic species prefer a less humid microclimateand a larger hollow entrance is associated with a reduction in humidity inside the tree hollow (Schauer et al., 2018).

We also found a positive relationship between hollow volume and number of threatened species in all three forest regions combined, as well as in Ebrach and Kelheim alone. As many threatened saproxylic beetle species are assumed to have high habitat requirements concerning not only quantity but quality of dead wood structures (Müller et al., 2005), several studies have reported the association of threatened species with large tree hollows that offer a greater diversity of microhabitats (Ranius et al., 2009).

There was a negative relationship between total species richness and the stage of wood mould decomposition when all three management districts were combined as well as for Ebrach alone for all species as well as for threatened species. A similar relationship has been reported by Stokland and Siitonen (2012), who proposed that the number of saproxylic species in forest dead wood declined at later stages of decay. While we found early stages of wood mould decomposition to contain the highest species richness, both Sverdrup‐Thygeson et al. (2010) and Schauer et al. (2018) reported the highest species richness in tree hollows with intermediate stages of wood mould decomposition.

In Ebrach total species richness of saproxylic beetles was related to temperature inside the tree hollows. We found the highest species richness in tree hollows with intermediate mean temperatures of 17–18°C. The positive relationship between temperature and species richness in the *r* = 500 m model can be explained by three hollows with a relatively high average temperature that contained relatively few beetle species and that were randomly removed from the *r* = 500 m model due to spatial overlap with other hollows or non‐forest areas. However, for threatened species in all three forest regions combined, as well as in Kelheim alone, we observed a strictly positive relationship with temperature. Many other studies also reported a positive relationship between temperature and species richness (Müller et al., 2015; Schauer et al., 2018), especially for threatened saproxylic beetle species (Lindhe et al., 2005; Widerberg et al., 2012).

In Ebrach and Kelheim we found higher species richness in tree hollows located closer to the ground. We observed the same relationship for total species richness in all three forest regions combined as well as for threatened species in Ebrach. This has also been reported by Schauer et al. (2018) for the Ebrach management district as well as Quinto et al. (2014) in Mediterranean deciduous oak woodland. One possible explanation could be the higher number of generalist predator species in tree hollows located closer to the ground (Ranius, 2002; Ranius, Svensson, et al., 2009). It has also been reported that generalist species that can dwell on the forest floor are more dominant in tree hollows that are connected to the ground (Siitonen, 2012). However, as we only examined tree hollows up to a height of 350 cm above ground, we only cover a limited vertical range of tree hollows, which may bias results.

Total and threatened species richness for all three management districts combined was negatively related to height of the tree hollows above sea level. It has previously been shown that altitude negatively affected saproxylic beetle diversity (Johansson et al., 2017; Wu et al., 2008) as higher elevations are generally associated with colder climate. Johansson et al. (2017) described gradients in altitude as important determinants of the distribution of saproxylic beetle assemblages in boreal forests in Sweden. Similarly, Müller et al. (2015) proposed that conservationists, landscape managers, and ecologists should pay more attention to the climate gradient as one fundamental driver of saproxylic insect diversity.

### 4.2 Influence of forest structure at larger spatial scales

Beside parameters of the tree hollows themselves, we also analyzed forest structure at different spatial scales. In contrast to tree hollow parameters, the parameters of forest structure that influenced saproxylic beetle diversity in tree hollows were mostly highly idiosyncratic with respect to region, which was most likely due to the differences in tree‐species composition between regions.

It has previously been reported that the number of tree hollows in the surrounding of a focal tree hollow had a positive influence on species richness of saproxylic beetles (Ranius & Wilander, 2000; Schauer et al., 2018). In our study we could not confirm this, but the number of microhabitat structures, indicating the number of possible future tree hollows or other dead wood related structures to develop, in a 30 m radius around each focal tree hollow explained species richness of saproxylic beetles across all three forest regions as well as for Ebrach and Kelheim alone. The same result was obtained for threatened species in all three regions combined as well as for Ebrach and Fichtelberg alone.

In Ebrach, the region with the highest proportion of deciduous tree species, species richness of saproxylic beetles showed a significant positive relationship with dead wood volume at small radii of 50 and 100 m around the focal tree hollows but not at larger spatial scales. A reason for the restriction of the influence of dead wood volume to smaller spatial scales might be that dispersal abilities of most saproxylic insect species are still unknown and might be rather small (Feldhaar & Schauer, 2018). In the other two forest management districts, with fewer beech trees and a lower proportion of deciduous trees, there was no relationship between dead wood volume and species richness. The importance of dead wood for a diverse fauna of saproxylic insects in forests has been extensively described before (Floren et al., 2014; Gossner, Floren, et al., 2013; Müller et al., 2015; Similä et al., 2003). Lassauce et al. (2011) proposed that additional variables like the type of dead wood or stage of decomposition should be taken into account. Stokland and Siitonen (2012) state that the majority of saproxylic beetle species (89%) are specialized either on deciduous or on coniferous dead wood. Dead wood of mostly coniferous trees, as it prevailed in the forest management district Fichtelberg, did not promote species richness of saproxylic beetles in tree hollows in beech trees. In contrast, the only parameter that influenced total species richness of saproxylic beetles in the conifer dominated Fichtelberg management district was the proportion of beech trees in the surrounding of focal tree hollows, up to a radius of 100 m around tree hollows. This finding implies that in a forest region like the Fichtelberg management district where suitable tree hollow habitats in deciduous trees are very limited the number of potential habitat trees in the surrounding of a focal tree hollow is the most important parameter influencing saproxylic beetle diversity in tree hollows, and the quality of the habitat – the tree hollow itself – might be of secondary importance.

Implications for forest management that can be inferred from this study include the conservation of developing and existing tree hollows as each tree hollow is unique with regard to the range of microhabitats within (Quinto et al., 2014; Siitonen, 2012). This study has also shown that the number of tree‐related microhabitats (e. g. woodpecker holes, injuries to the bark) in a forest stand is not only beneficial to the development of future tree hollows but seems to already increase species richness of saproxylic beetles in tree hollows and should therefore be conserved. Another important implication for forest management is the further enrichment of dead wood in managed forests (Gossner, Floren, et al., 2013; Müller et al., 2015), especially dead wood from deciduous tree species as saproxylic beetles have been shown to react sensitively not only to the amount but also the variety and distribution of dead wood (Sverdrup‐Thygeson et al., 2014). Tree‐species composition has also proven to greatly influence diversity of hollow‐using beetles (Floren et al., 2014). Therefore, increasing the proportion of deciduous trees in managed forests with a high proportion of coniferous trees will increase species richness of saproxylic beetles in tree hollows.

## 5 CONCLUSIONS

Local tree hollow characteristics influence the diversity of saproxylic beetles in tree hollows irrespective of region and management. Parameters of forest structure at larger spatial scales differ in their importance for saproxylic beetle species richness depending on the forest region's tree‐species composition. Therefore, the influence of local tree hollow parameters on species richness of saproxylic beetles should be considered in the context of surrounding forest structure at larger spatial scales. Forest inventory data can support this process by providing necessary data without additional field work. Thus, forest inventory data can be a powerful tool, in combination with local tree hollow parameters, to assess diversity of hollow‐using beetles in managed forests and help forest authorities decide which conservation measures to apply in certain parts of forest regions to effectively protect saproxylic beetles in tree hollows.

## CONFLICT OF INTEREST

The authors declare that there are no conflicts of interest.

## AUTHOR CONTRIBUTIONS

**Benjamin Henneberg:** Conceptualization (equal); Data curation (lead); Formal analysis (lead); Investigation (equal); Methodology (equal); Software (equal); Supervision (equal); Visualization (lead); Writing‐original draft (lead); Writing‐review & editing (lead). **Simon Bauer:** Data curation (equal); Formal analysis (equal); Investigation (equal); Writing‐original draft (supporting). **Markus Birkenbach:** Data curation (equal); Formal analysis (equal); Investigation (equal); Writing‐original draft (supporting). **Vanilla Mertl:** Data curation (equal); Formal analysis (equal); Investigation (equal); Writing‐original draft (supporting). **Manuel J. Steinbauer:** Data curation (equal); Formal analysis (equal); Methodology (equal); Software (equal); Visualization (supporting). **Heike Feldhaar:** Conceptualization (lead); Funding acquisition (lead); Methodology (lead); Project administration (lead); Supervision (lead); Writing‐original draft (supporting). **Elisabeth Obermaier:** Conceptualization (lead); Funding acquisition (lead); Methodology (lead); Project administration (lead); Supervision (lead); Writing‐original draft (supporting).

## Data Availability

The data supporting the findings of this study are available from Dryad digital repository: https://doi.org/10.5061/dryad.66t1g1k1q.

## APPENDIX A

**TABLE A1:** Multivariate GLMs of the Ebrach management district (2018) of different radii around the focal tree hollows (*r* ≤ 100 m: *n* = 50, *r* = 200 m: *n* = 37, *r* = 300 m: *n* = 32, *r* = 500 m: *n* = 24)

GLM/radius	Height above ground		Stage of decomposition		Temperature		Microhabitat structures		Dead wood volume (FI)		Pseudo‐*R* ^2^
*r* = 0 m	n. s.		*p* = .006**	*z* = −2.746	*p* = .001**	*z* = 3.220	n. a.		n. a.		.378
*r* = 50 m	*p* = .021*	*z* = −2.313	*p* = .005**	*z* = −2.824	*p* = .0001***	*z* = 3.821	n. s.		*p* = .001**	*z* = 3.238	.507
*r* = 100 m	*p* = .019*	*z* = −2.343	*p* = .014*	*z* = −2.446	*p* = .0001***	*z* = 3.799	n. s.		*p* = .007**	*z* = 2.717	.480
*r* = 200 m	n. s.		*p* = .0003***	*z* = −3.584	*p* = .002**	*z* = 3.162	*p* = .021*	*z* = 2.317	n. s.		.457
*r* = 300 m	n. s.		*p* = 9.86e−06***	*z* = −4.420	*p* = 2.91e−05***	*z* = 4.180	*p* = .033*	*z* = 2.136	n. s.		.595
*r* = 500 m	*p* = .030*	*z* = −2.165	*p* = .015*	*z* = −2.435	*p* = .003**	*z* = 3.003	n. s.		n. s.		.740

Note

Total species richness of saproxylic beetles as dependent variable; *p* < .05 (*), *p* < .01 (**), *p* < .001 (***).

Abbreviations: n. a., not available; n. s., not significant.

**TABLE A2:** Multivariate GLMs of the Ebrach management district (2018) of different radii around the focal tree hollows (*r* ≤ 100 m: *n* = 50, *r* = 200 m: *n* = 37, *r* = 300 m: *n* = 32, *r* = 500 m: *n* = 24)

GLM/radius	Hollow volume		Height above ground		Stage of decomposition		Microhabitat structures		Pseudo‐*R* ^2^
*r* = 0 m	*p* = .008**	*z* = 2.648	*p* = .017*	*z* = −2.383	*p* = .028*	*z* = −2.198	n. a.		.288
*r* = 50 m	n. s.		*p* = .003**	*z* = −2.989	*p* = .048*	*z* = −1.980	*p* = .001**	*z* = 3.214	.438
*r* = 100 m	n. s.		*p* = .004**	*z* = −2.918	n. s.		*p* = .001**	*z* = 3.248	.443
*r* = 200 m	n. s.		*p* = .015*	*z* = −2.441	*p* = .005**	*z* = −2.780	*p* = .011*	*z* = 2.554	.386
*r* = 300 m	*p* = .009**	*z* = 2.577	*p* = .011*	*z* = −2.541	n. s.		*p* = .015*	*z* = 2.428	.496
*r* = 500 m	n. s.		*p* = .014*	*z* = −2.468	*p* = .026*	*z* = −2.234	*p* = .019*	*z* = 2.349	.691

Note

Species richness of threatened saproxylic beetles as dependent variable; *p* < .05 (*), *p* < .01 (**), *p* < .001 (***).

Abbreviations: n. a., not available; n. s., not significant.

**TABLE A3:** Multivariate GLMs of the Fichtelberg management district (2018) of different radii around the focal tree hollows (*r* ≤ 100 m: *n* = 43, *r* = 200 m: *n* = 36, *r* = 300 m: *n* = 33, *r* = 500 m: *n* = 28)

GLM/radius	Proportion of beech trees (FI)		Pseudo‐*R* ^2^
*r* = 0 m	n. a.		
*r* = 50 m	*p* = .023*	*z* = 2.269	.113
*r* = 100 m	*p* = .023*	*z* = 2.280	.116
*r* = 200 m	n. s.		
*r* = 300 m	n. s.		

Note

Total species richness of saproxylic beetles as dependent variable; *p* < .05 (*), *p* < .01 (**), *p* < .001 (***).

Abbreviations: n. a., not available; n. s., not significant.

**TABLE A4:** Multivariate GLMs of the Fichtelberg management district (2018) of different radii around the focal tree hollows (*r* ≤ 100 m: *n* = 43, *r* = 200 m: *n* = 36, *r* = 300 m: *n* = 33, *r* = 500 m: *n* = 28)

GLM/radius	Height above sea level		Microhabitat structures		Proportion of beech trees (FI)		Pseudo‐*R* ^2^
*r* = 0 m	*p* = .004**	*z* = −2.889	n. a.		n. a.		.287
*r* = 50 m	n. s.		*p* = .028*	*z* = 2.196	*p* = .011*	*z* = 2.540	.487
*r* = 100 m	n. s.		*p* = .027*	*z* = 2.211	*p* = .012*	*z* = 2.501	.483
*r* = 200 m	n. s.		*p* = .021*	*z* = 2.306	n. s.		.304
*r* = 300 m	n. s.		n. s.		n. s.		.203

Note

Species richness of threatened saproxylic beetles as dependent variable; *p* < .05 (*), *p* < .01 (**), *p* < .001 (***).

Abbreviations: n. a., not available; n. s., not significant.

**TABLE A5:** Multivariate GLMs of the Kelheim management district (2019) of different radii around the focal tree hollows (*r* ≤ 100 m: *n* = 41, *r* = 200 m: *n* = 35, *r* = 300 m: *n* = 32)

GLM/radius	Area of hollow entrance		Height above ground		Microhabitat structures		Pseudo‐*R* ^2^
*r* = 0 m	*p* = .034*	*z* = −2.121	*p* = .042*	*z* = −2.035	n. a.		.240
*r* = 50 m	*p* = .049*	*z* = −1.968	n. s.		*p* = .002**	*z* = −3.155	.434
*r* = 100 m	*p* = .035*	*z* = −2.105	n. s.		*p* = .002**	*z* = −3.145	.427
*r* = 200 m	*p* = .026*	*z* = −2.225	*p* = .050*	*z* = −1.963	*p* = .001**	*z* = −3.190	.388
*r* = 300 m	*p* = .024*	*z* = −2.255	*p* = .047*	*z* = −1.985	*p* = .0007***	*z* = −3.408	.424

Note

Total species richness of saproxylic beetles as dependent variable; *p* < .05 (*), *p* < .01 (**), *p* < .001 (***).

Abbreviations: n. a., not available; n. s., not significant.

**TABLE A6:** Multivariate GLMs of the Kelheim management district (2019) of different radii around the focal tree hollows (*r* ≤ 100 m: *n* = 41, *r* = 200 m: *n* = 35, *r* = 300 m: *n* = 32)

GLM/radius	Area of hollow entrance		Hollow volume		Temperature		Pseudo‐*R* ^2^
*r* = 0 m	*p* = .011*	*z* = 2.542	*p* = .001**	*z* = 3.210	*p* = .022*	*z* = 2.287	.293
*r* = 50 m	*p* = .011*	*z* = 2.542	*p* = .001**	*z* = 3.210	*p* = .022*	*z* = 2.287	.293
*r* = 100 m	*p* = .011*	*z* = 2.542	*p* = .001**	*z* = 3.210	*p* = .022*	*z* = 2.287	.293
*r* = 200 m	*p* = .011*	*z* = 2.542	*p* = .001**	*z* = 3.210	*p* = .022*	*z* = 2.287	.293
*r* = 300 m	*p* = .011*	*z* = 2.542	*p* = .001**	*z* = 3.210	*p* = .022*	*z* = 2.287	.293

Note

Species richness of threatened saproxylic beetles as dependent variable; *p* < .05 (*), *p* < .01 (**), *p* < .001 (***).

Abbreviations: n. a., not available; n. s., not significant.

**TABLE A7:** Generalized linear mixed‐effects models (GLMEs) of the three forest management districts combined (*n* = 134 tree hollows)

	Random factor: Ebrach	Random factor: Fichtelberg	Random factor: Kelheim	Height above sea level		Area of hollow entrance		Height above ground		Stage of decomposition		Microhabitat structures	
GLME total spp.	Estimate = 0.187	Estimate = −0.104	Estimate = −0.080	*p* = 8.41e−06***	*z* = −4.454	*p* = 3.08e−05***	*z* = 4.168	*p* = .032*	*z* = −2.145	*p* = .028*	*z* = −2.193	*p* = .007**	*z* = 2.701

Note

Total species richness of saproxylic beetles as dependent variable; *p* < .05 (*), *p* < .01 (**), *p* < .001 (***).

Abbreviations: n. a., not available; n. s., not significant.

**TABLE A8:** Generalized linear mixed‐effects models (GLMEs) of the three forest management districts combined (*n* = 134 tree hollows)

	Random factor: Ebrach	Random factor: Fichtelberg	Random factor: Kelheim	Height above sea level		Hollow volume		Temperature		Microhabitat structures	
GLME threatened spp.	Estimate = 2.741e−16	Estimate = −1.844e−16	Estimate = −8.970e−17	*p* = 4.61e−08***	*z* = −5.466	*p* = .029*	*z* = 2.180	*p* = 7.99e−08***	*z* = 5.367	*p* = .045*	*z* = 2.007

Note

Species richness of threatened saproxylic beetles as dependent variable; *p* < .05 (*), *p* < .01 (**), *p* < .001 (***).

Abbreviations: n. a., not available; n. s., not significant.
